# Structural Damage Identification Based on Rough Sets and Artificial Neural Network

**DOI:** 10.1155/2014/193284

**Published:** 2014-06-11

**Authors:** Chengyin Liu, Xiang Wu, Ning Wu, Chunyu Liu

**Affiliations:** ^1^Harbin Institute of Technology, Shenzhen Graduate School, Shenzhen 518055, China; ^2^Key Laboratory of C&PC Structures, Southeast University, Nanjing 211189, China; ^3^State Key Laboratory of Robotics and System, Harbin Institute of Technology, Dazhi Street, Nangang District, Harbin 150001, China

## Abstract

This paper investigates potential applications of the rough sets (RS) theory and artificial neural network (ANN) method on structural damage detection. An information entropy based discretization algorithm in RS is applied for dimension reduction of the original damage database obtained from finite element analysis (FEA). The proposed approach is tested with a 14-bay steel truss model for structural damage detection. The experimental results show that the damage features can be extracted efficiently from the combined utilization of RS and ANN methods even the volume of measurement data is enormous and with uncertainties.

## 1. Introduction

Structures are very vulnerable to influence like impact, earthquake and hurricanes. Therefore it is crucial for the decision maker to know the damage and health status of the structure in time, so that necessary maintenance can be taken. Recently, more and more innovative structural damage detection techniques have been applied to the existing structures for Structural Health Monitoring (SHM), especially large-scale structures, and many testing methods are nondestructive [1–3]. Attention has been drawn to how to use the current measurement data to produce a result with less uncertainty regardless of measurement noises and environmental variation, such as changing temperature, moisture, and load condition [4]. Many different approaches have been applied to solve the inaccurate measurement problem, for example, Sohn et al. proposed a probabilistic damage detection methodology to reduce measurement noises [5]. Worden and Dulieu-Barton investigated the influence of uncertainties both in practical measurement and in finite element model of damage detection [6], and they proposed a statistical method to resolve the inaccuracy that resulted from the modeling and measurement errors [7]. In recent studies, intelligent information processing techniques such as the autoregressive integrated moving average model, linear regression technique, ANN methods, and grey models are introduced to SHM applications.

ANN methods have been used extensively in structural damage identification. In practice, damage indexes in structures are firstly extracted by using signal processing techniques such as wavelet transform and Fourier analysis; then ANN models are built to detect structural damages from those indexes. It has been widely accepted that the ANN methods have helped to achieve a greater accuracy in structural damage detection. However, ANN has two obvious drawbacks when applying to a large number of data [8, 9]. The first one is that training an ANN model with big amount of data is time consuming, and the second one is that ANN cannot reach an analytical solution. In consequence, a reliable ANN model that can select the relevant factors automatically from the historical data is required.

As a useful mathematical tool, RS theory applies the unclear relation and data pattern comparison based on the concept of an information system with indiscernible data, where the data is uncertain or inconsistent. The characteristics of RS theory are to create approximate descriptions of objects for data analysis, optimization, and recognition, and it does not need the prior knowledge. Therefore using RS theory can evaluate the importance of various attributes and retain some key attributes with no additional knowledge except for the supplied data required [10]. To date, the RS approach has been applied in many domains, such as machine fault diagnosis, stock market forecast, decision support systems, medical diagnosis, data filtration, and software engineering [11–14].

The classical RS model can only be used to process categorical features with discrete values. For the RS based damage index selection in structural damage identification, a discretizing algorithm is required to partition the value domains of real-valued variables into several intervals as categorical features. Many discretization methods of numerical attributes have been proposed in recent years, including equal distance method, equal frequency method, and maximum entropy method [9]. However, discretization of numerical attributes may cause information loss because the degrees of membership of numerical values to discretized values are not considered [15, 16]. Recently, a discretization algorithm based on information entropy has been reported to be a potential mechanism for the measurement of uncertainty in RS. The information entropy has been widely employed in RS, and different information entropy models have been proposed. In particular, Düntsch and Gediga presented a well-justified information entropy model for the measurement of uncertainty in RS [17].

A novel application of integrating RS theory and ANN is presented in this paper for structural health monitoring and damage detection particularly for problems with large measurement data with uncertainties. The objective of the paper is to study how the RS and ANN techniques can be combined to detect structural damages. This method consists of three stages. First, RS will be applied to find relevant factors for structural modal parameters derived from structural vibration responses. Then, relevant information will be fed to the ANN as input. Finally, a synthesizing RS-ANN model based on the data-fusion technique will be used to assess the structural damage.

This paper is organized as follows. In Section 2, a brief introduction of fundamental theories on RS with information entropy is presented, and an overview of the ANN methods is given in Section 3. A three-stage damage detection model using combined RS and ANN technique is presented in Section 4. Laboratory experiment of a 14-bay truss model will be carried out to test and validate the proposed method in Section 5. Finally, concluding remarks are summarized in Section 6.

## 2. Information Entropy Based RS Theory

RS theory was proposed by Pawlak [18] as a new mathematical tool for reasoning about vagueness, uncertainty, and imprecise information. In this section, we introduce the concepts of decision table, discretization algorithm, and information entropy in RS theory and explain their relationships.

### 2.1. RS Theory

We have the following.

**Definition 1**

Decision table is a knowledge representation system in the application of RS theory with a quaternary (*X*, *R*, *V*, *f*) set, where *X* is a set of targets, and *R* is a set of attributes, *R* = *C* ∪ *D*. *C* and *D* are condition attribute set and decision attribute set, respectively. *V* = ∪*V*_*r*_ is a set of attributes' data range. *V*_*r*_ is the range of attribute *r*. *f* : *X* × *R* → *V*; *f* is an information function, which assigns the range of each attribute.  Table 1 is a typical decision table.

**Definition 2**

*X* is a domain of discourse. *P* and *Q* are equivalence relations of universe *X*; then the *P*-positive region of *Q* is defined by the union of all the objects of *U* which can be classified as the equivalence class of *U*/*Q* by the knowledge *U*/*P*; that is,

(1) POSP(Q)=⋃Z∈X/QP(Z).

**Definition 3**

Let *P* and *Q* be equivalence relations of *U*. If (2) is satisfied, then *r* ∈ *P* is said to be *Q*-dispensable in *P*; otherwise, *r* ∈ *P* is *Q*-indispensable in *P*. If all *r* are *Q*-indispensable in *P*, *P* is said to be independent with respect to *Q*. Consider

(2) POSP(Q)=POSP−{r}(Q).

**Definition 4**

If *S*⊆*P* is *P*-independent and POS_*S*_(*Q*) = POS_*P*_(*Q*) is satisfied, then *S* is said to be the *Q*-reduct of *P*, that is, RED_*Q*_(*P*), and the union of all the *Q*-indispensable attributes is said to be the *Q*-core of *P*, that is, CORE_*Q*_(*P*). The relation of these two notions is expressed as

(3) COREQ(P)=∩REDQ(P).

### 2.2. Discretization Algorithm Based on Information Entropy

Let *U*⊆*X* be a subset, and the number of instances is |*U*|. The number of* j*th (*j* = 1,2,…, *r*) decision attribute is *k*_*j*_. Let the information entropy of this subset be

(4) H(U)=−∑j=1r(d)pjlog2pj, pj=kj|U|.

In general, *H*(*U*) ≥ 0. If the information entropy is small, it reveals that several decision attributes are predominant, and the complexity is small. All the decision attributes especially are the same, and *H*(*U*) = 0. For the breakpoint *c*_*i*_^*a*^ in the example, its decision attribute is *j*  (*j* = 1,2,…, *r*); the number of decision attributes less than *c*_*i*_^*a*^ in the set *U* is *l*_*j*_^*U*^(*c*_*i*_^*a*^), and the number of decision attributes greater than *c*_*i*_^*a*^ in the set *U* is *r*_*j*_^*U*^(*c*_*i*_^*a*^). Let

(5) lU(cia)=∑j=1r(d)ljU(cia),rU(cia)=∑j=1r(d)rjU(cia).

Therefore the breakpoint *c*_*i*_^*a*^ could divide the set *U* into two subsets *X*_*l*_ and *X*_*r*_. Let

(6) H(Xl)=−∑j=1r(d)pjlog2pj, pj=ljU(cia)lU(cia),H(Xr)=−∑j=1r(d)qjlog2qj, qj=rjU(cia)rU(cia).

The information entropy of the breakpoint *c*_*i*_^*a*^ to the set *U* is rewritten as

(7) HU(cia)=|Xl||X|H(Xl)+|Xr||X|H(Xr).

Assume that *L* = {*Y*_1_, *Y*_2_,…, *Y*_*m*_} is the equivalence selected by decision table; the new information entropy of the new breakpoint *c* ∉ *P* can be written as

(8) H(c,L)=HY1(c)+HY2(c)+⋯+HYm(c).

Let *P* be the set of the chosen breakpoints, *L* is an equivalent set divided by breakpoint set *P*, *S* is the set of the initial breakpoint, and *H* is the information entropy of decision table; our discretization algorithm can be expressed as follows.

**Step 1**

*P* = *∅*; *L* = {*X*}; *H* = *H*(*X*).

**Step 2**

To any *c* ∈ *S*, calculate *H*(*c*, *L*).

**Step 3**

If *H* ≤ min⁡{*H*(*c*, *L*)}, go to the end.

**Step 4**

Select *c*_max⁡_ into *P* to make *H*(*c*, *L*) be minimum, *H* = *H*(*c*, *L*)*S* = *S* − {*c*}.

**Step 5**

To all *U* ∈ *L*, if *c*_max⁡_ divide the equivalence *U* into *X*_1_ and *X*_2_, then delete *U* from *L* and join the equivalence *X*_1_ and *X*_2_ into *L*.

**Step 6**

If any equivalence in *L* has the same decision, go to the end. Otherwise go to Step 2.

## 3. Artificial Neural Network (ANN)

An artificial neural network (ANN) is an information processing paradigm inspired by biological nervous systems like brains. Although ANNs model the mechanism of brain, they do not have analytical function form, and therefore ANNs are data based instead of model based. An ANN is usually composed of a large number of highly interconnected processing elements (neurons) working in unison to solve specific problems.

The ANN used in this study is arranged in three layers of neurons, namely, the input, hidden, and output layers. The input layers introduce the model inputs, and the middle layer of hidden units feeds into an output layer through variable weight connections. The ANN learns by adjusting the values of these weights through a back-propagation algorithm that permits error corrections to be fed through the layers. Output layer provides the estimations of the network. An ANN is renowned for their ability to learn and generalize from example data, even when the data is noisy and incomplete. This ability has led to an investigation into the application of ANNs to automated knowledge acquisition. They also help to discern patterns among input data, require fewer assumptions, and achieve a higher degree of prediction accuracy.

## 4. The Hybrid Method

The common advantage of RS and ANN is that they do not need any additional information about data like probability in statistics or grade of membership in fuzzy-set theory [19]. RS has proved to be very effective in many practical applications. However, in RS theory, the deterministic mechanism for the description of error is too straightforward [20], and therefore the rules generated by RS are often unstable and have low classification accuracies. In consequence, RS cannot identify structural damage with a high accuracy. ANN is generally considered to be the most powerful classifier for low classification-error rates and robustness to noise. The knowledge of ANN is buried in their structures and weights [21, 22]. It is often difficult to extract rules from a trained ANN. The combination of RS and ANN is very natural for their complementary features.

One typical approach is to use the RS approach as a preprocessing tool for the ANN [12, 23]. RS theory provides useful techniques to reduce irrelevant and redundant attributes from a large database with various attributes. ANN has the ability to approach any complex functions and possess a good robustness to noise. In practice, there are often vast amounts of sensor data that are typically updated every few minutes in SHM system. One of the most important issues of RS theory is the reduction in dimension of the decision table in terms of both attributes and objects, thereby reducing the redundancy.

This paper will develop the structural damage model by using the RS methodology to reduce the dimension of the structural damage database before applying the ANN method. Firstly, the following reductions can be derived based on the RS theory: attribute reduction, object reduction, and rule generation. Object reduction involves reducing the rows of the database in terms of redundant objects (rows). Rule generation involves the generation of If-Then rules from the database. Then the ANN is trained to learn in order to predict the damage conditions.

## 5. Experimental Validation

### 5.1. Test Structure

**Figure 1:**
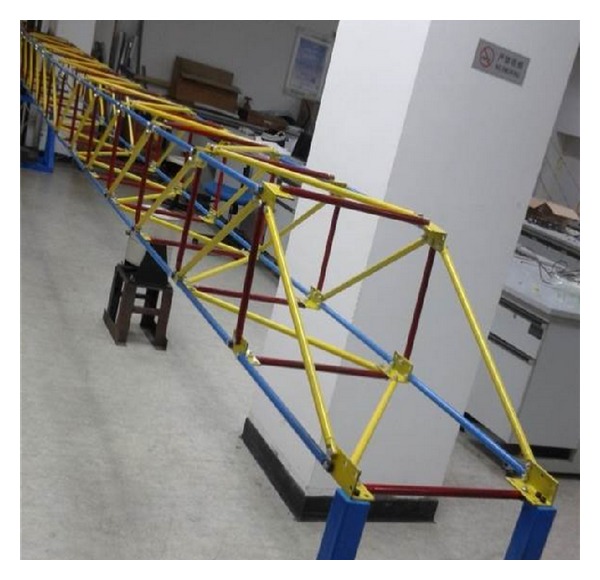
Test structure.

The test structure is a steel truss with 14 bays, shown in Figure 1. Each bay is 585 mm long, 490 mm wide, and 350 mm high. Totally, the steel truss has 52 longitudinal rods, 50 crosswise rods, and 54 diagonal rods. Each rod is forged with steel pipe. The section of the rods is hollow circular with an outer diameter of 18 mm, and inner diameter of 12 mm. Node board uses equilateral angle steel. Rods are bolted on the node board. Damages of the structure are simulated by two kinds of reduced thickness rods. One is 2 mm thick, and the other is 1 mm thick.

**Figure 2:**
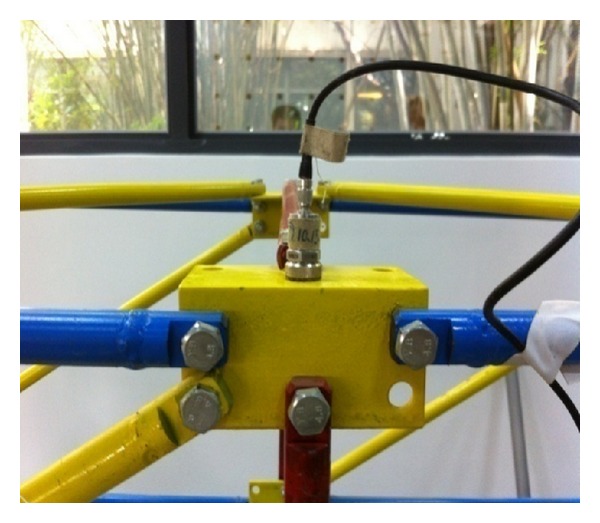
Accelerometer.

Accelerometers are mounted on each node of the structure as shown in Figure 2. The sampling interval of measurements retrieved from the data acquisition system is 5 min.

### 5.2. Establishment of Damage Database

**Figure 3:**
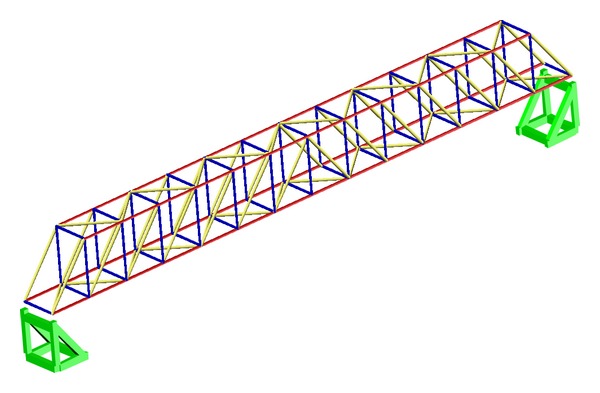
Test structure FE model.

A FE model was built to simulate the test structure as shown in Figure 3. In this study, three types of damage conditions are investigated, respectively, including damage bay, damage position, and damage degree. Since the end bays have no upper rod, the damage bay starts from the second span. Thus 12 bays are assumed to be damaged. In these bays, damage positions in upper rod, diagonal rod, and bottom rod are all known. For damage degree, we simulate the stiffness from 95% to 5% with the interval of 5%. In total there are 19 different kinds of damage degrees. Combining these three damage conditions, we have 684 damage conditions in total.

According to the FEA results, 13 structural damage indexes are extracted, including the first three natural frequencies, the first three strain modes, the first three vibration mode shapes, modal assurance criterion (MAC), coordinate modal assurance criterion (COMAC), curvature mode, and natural frequency square. These indexes, together with damage conditions, form a 684 rows and 124 columns structural damage database (decision table) in this study.  Table 2 lists part of the database. Note that in the damage position column, number 1, 2, and 3 represent the upper rod, diagonal rod, and bottom rod, respectively.

### 5.3. Attribute Reduction

In this section, application of RS to data reduction involves three steps (see below).

#### 5.3.1. Step 1: Reduction of Decision Table

The damage database is reduced in batches as shown in Tables 3, 4, and 5. From the reduced database, it can be seen that the data volume has been greatly reduced. The core of the database is the first three natural frequencies. In order to ensure the integrity of the damage indexes, less reduced condition attributes are remained. There are 3 minimum properties in total. They are the first three frequencies with the first order strain mode (set 1), the first three frequencies with the second order strain mode (set 2), and the first three frequencies with the third order strain mode (set 3), respectively.

#### 5.3.2. Step 2: Discretization of Reduced Decision Table

Through the discretization of the three attribute sets, a set of reduced decision tables can be obtained. The attribute sets (1, 2, and 3) are discretized according to the decision attributes, the damage bay (DB), and the damage position (DP), respectively.

Table 6 summarizes the intervals of each decision attribute resulted from the discretization of the three attribute sets. It is found that, for the decision attribute of damage bay, the intervals are much more in the strain mode condition attributes than those in natural frequency condition attributes. While for the decision attribute of damage position, the intervals are much more at the natural frequency condition attributes than those in strain mode condition attributes. The result demonstrates that the strain mode has more weights in identification of structural damage bay, while the natural frequency has more weights in identification of structural damage position.

#### 5.3.3. Step 3: Rules Generation

Rules generation is a key step in the RS analysis. In this study, the rules are generated from the discretized decision table in the form of knowledge. According to the exclusive rule extraction method, the same condition and decision attributes are removed. Therefore, simplified decision tables are obtained as shown in Tables 7, 8, 9, 10, 11, and 12. These decision tables demonstrate that every single damage case is unique.

From Table 7 to Table 12, it can be seen that the rows of each table are decreased to less than half of the original ones after rules generation. Each attribute set has its own rule of damage identification. The values of rule generation result for damage bay are less than those for damage position on average. It illustrates that the identification of damage bay is easier than that of damage position.

### 5.4. Identification of Structure Damage Using ANN

In this section, back-propagation ANN is applied to the reduced database for further identification of structural damages. The reduced database in terms of attributes can be described as the best subset of variables which describe the structural damage database completely. This reduction in number of attributes decreases the time of decision-making process and consequently reduces the cost of efficiency analysis. As mentioned above, three attribute sets are chosen as the input, and three damage conditions are chosen as the output to train the ANN model. The back-propagation network computes the weights in a recurrence mode from the last layer backward to the first layer.

Using real data obtained from the experimental testing, we put the experimental measurements into the trained ANN input layer to identify the structural damage. The results in Tables 13, 14, and 15 show that the RS method determines the group of input variables and generates the structural damage rule sets before using ANN. While the performance of the ANN model on identification of damaged degree is not very good, the hybrid method proposed in the paper is helpful to construct a good identification model for structural damage, offering an excellent performance of identifying the damaged bay and damaged position of the test structure.

## 6. Conclusions

In this paper, a novel method of combining RS and ANN methods is applied to the identification of structural damages. This study uses RS theory and integrates the inductive reduction algorithm and discretization algorithm based on information entropy to improve the ANN model for structural damage identification. Through a detailed experimental analysis of a 14-bay truss structure, this paper presents and discusses the conversion of damage index to RS object, predicting variables selection, removal of redundant from information table, and rules generation. The experiments data is preprocessed and reduced by RS before using ANN for identifying the damages of truss structure. The identification accuracy is mainly attributed to RS since it can remove redundant attributes without any classification information loss. Furthermore, the improvement in tolerance and accuracy with the proposed method shows that there is a great potential for integration of various techniques to improve the performance of an individual technique.

## Figures and Tables

**Table 1:** Decision table general form.

Target	Condition attribute			Decision attribute
*X*	*C*_1_	*⋯*	*C*_*n*_	*d*
*x*_ 1_	*u*_1,1_	*⋯*	*u*_1,*n*_	*v*_1_
*x*_2_	*u*_2,1_	*⋯*	*u*_2,*n*_	*v*_2_
*⋯*	*⋯*	*⋯*	*⋯*	*⋯*
*x*_*m*_	*u*_*m*,1_	*⋯*	*u*_*m*,*n*_	*v*_*m*_

**Table 2:** Structural damage database.

Damage case	Damage condition			Natural frequency			*⋯*	Mode curvature		
	Bay	Position	Degree	1	2	3	*⋯*	1	*⋯*	13
1	2	1	5%	8.76	32.34	61.57	*⋯*	0.006	*⋯*	0.006
2	2	1	10%	8.73	32.31	61.49	*⋯*	0.006	*⋯*	0.006
*⋯*	*⋯*	*⋯*	*⋯*	*⋯*	*⋯*	*⋯*	*⋯*	*⋯*	*⋯*	*⋯*
684	13	3	95%	7.84	24.53	52.32	*⋯*	0.006	*⋯*	0.018

**Table 3:** Minimum property set 1 after reduction.

Damage case	Damage condition			Natural frequency			First order strain mode		
	Span	Position	Degree	1	2	3	1	*⋯*	12
1	2	1	5%	8.76	32.34	61.57	0.003	*⋯*	0.003
2	2	1	10%	8.73	32.31	61.49	0.004	*⋯*	0.003
*⋯*	*⋯*	*⋯*	*⋯*	*⋯*	*⋯*	*⋯*	*⋯*	*⋯*	*⋯*
684	13	3	95%	7.84	24.53	52.32	0.003	*⋯*	0.018

**Table 4:** Minimum property set 2 after reduction.

Damage case	Damage condition			Natural frequency			Second order strain mode		
	Span	Position	Degree	1	2	3	1	*⋯*	12
1	2	1	5%	8.76	32.34	61.57	0.009	*⋯*	−0.006
2	2	1	10%	8.73	32.31	61.49	0.012	*⋯*	−0.006
*⋯*	*⋯*	*⋯*	*⋯*	*⋯*	*⋯*	*⋯*	*⋯*	*⋯*	*⋯*
684	13	3	95%	7.84	24.53	52.32	0.006	*⋯*	−0.036

**Table 5:** Minimum property set 3 after reduction.

Damage case	Damage condition			Natural frequency			Third order strain mode		
	Span	Position	Degree	1	2	3	1	*⋯*	12
1	2	1	5%	8.76	32.34	61.57	0.009	*⋯*	0.009
2	2	1	10%	8.73	32.31	61.49	0.015	*⋯*	0.009
*⋯*	*⋯*	*⋯*	*⋯*	*⋯*	*⋯*	*⋯*	*⋯*	*⋯*	*⋯*
684	13	3	95%	7.84	24.53	52.32	0.009	*⋯*	0.054

**Table 6:** Sections of each attribute set and condition attributes.

Damage case	Natural frequency			Strain mode											
	1	2	3	1	2	3	4	5	6	7	8	9	10	11	12
Set 1, DB	3	2	5	5	6	5	7	7	5	5	7	7	5	6	5
Set 2, DB	3	2	5	9	7	8	9	10	11	11	9	9	8	7	9
Set 3, DB	3	2	5	8	7	7	8	10	10	10	10	8	7	7	8
Set 1, DP	4	10	10	3	5	2	4	4	4	4	4	4	2	5	3
Set 2, DP	4	10	10	1	2	4	4	3	5	5	3	4	4	2	1
Set 3, DP	4	10	10	1	1	1	5	4	3	3	4	5	1	1	1

**Table 7:** Attribute set 1 rules generation for damage bay.

Damage case	Natural frequency			First order strain mode												
	1	2	3	1	2	3	4	5	6	7	8	9	10	11	12	
1	3	2	3	4	3	3	2	3	3	3	4	2	3	3	3	2
2	3	2	3	5	3	3	2	2	2	3	2	2	2	3	2	2
*⋯*	*⋯*	*⋯*	*⋯*	*⋯*	*⋯*	*⋯*	*⋯*	*⋯*	*⋯*	*⋯*	*⋯*	*⋯*	*⋯*	*⋯*	*⋯*	*⋯*
139	3	2	2	2	3	2	2	2	3	2	2	2	3	3	5	12
140	2	1	1	2	2	2	2	2	2	2	2	2	2	2	5	12

**Table 8:** Attribute set 2 rules generation for damage bay.

Damage case	Natural frequency			Second order strain mode												
	1	2	3	1	2	3	4	5	6	7	8	9	10	11	12	
1	3	2	3	1	1	2	3	2	3	8	6	6	6	5	6	2
2	3	2	3	1	2	2	3	2	3	8	6	6	6	5	6	2
*⋯*	*⋯*	*⋯*	*⋯*	*⋯*	*⋯*	*⋯*	*⋯*	*⋯*	*⋯*	*⋯*	*⋯*	*⋯*	*⋯*	*⋯*	*⋯*	*⋯*
194	3	1	1	6	5	6	6	7	7	3	3	4	3	3	1	12
195	2	1	1	6	5	6	6	7	7	3	3	5	3	4	1	12

**Table 9:** Attribute set 3 rules generation for damage bay.

Damage case	Natural frequency			Third order strain mode												
	1	2	3	1	2	3	4	5	6	7	8	9	10	11	12	
1	3	2	3	7	5	5	6	7	9	5	3	1	1	1	1	2
2	3	2	3	7	5	5	6	7	10	5	3	1	1	1	1	2
*⋯*	*⋯*	*⋯*	*⋯*	*⋯*	*⋯*	*⋯*	*⋯*	*⋯*	*⋯*	*⋯*	*⋯*	*⋯*	*⋯*	*⋯*	*⋯*	*⋯*
229	3	1	1	2	1	1	1	3	5	10	7	6	5	5	8	12
230	2	1	1	2	1	1	1	3	6	10	7	6	5	5	8	12

**Table 10:** Attribute set 1 rules generation for damage position.

Damage case	Natural frequency			First order strain mode												
	1	2	3	1	2	3	4	5	6	7	8	9	10	11	12	
1	3	10	8	2	3	1	2	2	2	2	2	2	1	3	1	1
2	3	9	7	3	1	1	2	2	2	2	2	2	1	1	1	1
*⋯*	*⋯*	*⋯*	*⋯*	*⋯*	*⋯*	*⋯*	*⋯*	*⋯*	*⋯*	*⋯*	*⋯*	*⋯*	*⋯*	*⋯*	*⋯*	*⋯*
251	1	2	1	1	1	1	1	1	1	1	1	1	1	1	3	3
252	1	1	1	1	1	1	1	1	1	1	1	1	1	1	3	3

**Table 11:** Attribute set 2 rules generation for damage position.

Damage case	Natural frequency			Second order strain mode												
	1	2	3	1	2	3	4	5	6	7	8	9	10	11	12	
1	3	10	8	1	1	1	2	1	1	3	1	3	3	1	1	1
2	3	9	7	1	1	1	2	1	1	3	1	3	3	1	1	1
*⋯*	*⋯*	*⋯*	*⋯*	*⋯*	*⋯*	*⋯*	*⋯*	*⋯*	*⋯*	*⋯*	*⋯*	*⋯*	*⋯*	*⋯*	*⋯*	*⋯*
253	1	1	1	1	1	3	3	1	3	1	1	2	2	1	1	3
254	1	1	1	1	1	3	3	1	3	1	1	3	3	1	1	3

**Table 12:** Attribute set 3 rules generation for damage position.

Damage case	Natural frequency			Third order strain mode												
	1	2	3	1	2	3	4	5	6	7	8	9	10	11	12	
1	3	10	8	1	1	1	4	4	3	3	1	1	1	1	1	1
2	3	9	7	1	1	1	4	4	3	3	1	1	1	1	1	1
*⋯*	*⋯*	*⋯*	*⋯*	*⋯*	*⋯*	*⋯*	*⋯*	*⋯*	*⋯*	*⋯*	*⋯*	*⋯*	*⋯*	*⋯*	*⋯*	*⋯*
273	3	2	3	1	1	1	1	1	3	3	4	4	1	1	1	3
274	2	2	3	1	1	1	1	2	3	3	4	4	1	1	1	3

**Table 13:** Damage identification by using attribute set 1.

Expectation	Bay	Position	Degree	Recognition	Bay	Position	Degree
1	7	upper	28.8%	1	7.32	1.28	15.96%
2	7	upper	62.2%	2	6.93	1.02	48.53%
3	7	diagonal	28.8%	3	7.23	1.97	30.68%
4	7	diagonal	62.2%	4	7.08	2.01	57.75%
5	7	bottom	28.8%	5	7.11	3.33	10.84%
6	7	bottom	62.2%	6	7.07	3.13	20.68%
7	5	upper	28.8%	7	5.12	1.17	40.52%
8	5	upper	62.2%	8	4.88	0.74	63.84%
9	5	diagonal	28.8%	9	4.53	1.45	82.56%
10	5	diagonal	62.2%	10	4.54	1.15	35.53%
11	5	bottom	28.8%	11	5.16	2.63	68.45%
12	5	bottom	62.2%	12	5.22	3.04	72.34%

**Table 14:** Damage identification by using attribute set 2.

Expectation	Bay	Position	Degree	Recognition	Bay	Position	Degree
1	7	upper	28.8%	1	6.24	1.34	13.41%
2	7	upper	62.2%	2	7.42	1.34	35.42%
3	7	diagonal	28.8%	3	7.14	1.84	42.52%
4	7	diagonal	62.2%	4	6.73	1.93	45.14%
5	7	bottom	28.8%	5	7.24	2.04	85.31%
6	7	bottom	62.2%	6	7.21	3.54	51.96%
7	5	upper	28.8%	7	4.76	1.42	45.15%
8	5	upper	62.2%	8	4.62	0.67	13.56%
9	5	diagonal	28.8%	9	5.25	2.02	41.08%
10	5	diagonal	62.2%	10	5.11	1.44	68.28%
11	5	bottom	28.8%	11	5.62	3.52	49.31%
12	5	bottom	62.2%	12	6.21	3.13	25.19%

**Table 15:** Damage identification by using attribute set 3.

Expectation	Bay	Position	Degree	Recognition	Bay	Position	Degree
1	7	upper	28.8%	1	7.22	1.17	58.74%
2	7	upper	62.2%	2	5.82	0.74	20.84%
3	7	diagonal	28.8%	3	6.81	1.88	66.68%
4	7	diagonal	62.2%	4	7.03	2.35	59.52%
5	7	bottom	28.8%	5	6.81	2.63	60.39%
6	7	bottom	62.2%	6	7.59	3.04	63.84%
7	5	upper	28.8%	7	4.84	1.14	62.56%
8	5	upper	62.2%	8	5.04	1.36	83.15%
9	5	diagonal	28.8%	9	5.14	2.44	39.16%
10	5	diagonal	62.2%	10	5.15	1.97	43.18%
11	5	bottom	28.8%	11	4.91	3.74	25.44%
12	5	bottom	62.2%	12	4.70	3.02	72.82%

## References

[1] Farrar CR, Doebling SW, Nix DA (2001). Vibration-based structural damage identification. *Philosophical Transactions of the Royal Society A: Mathematical, Physical and Engineering Sciences*.

[2] Doebling SW, Farrar CR (1999). The state of the art in structural identification of constructed facilities.

[3] Doebling SW, Farrar CR, Prime MB (1998). A summary review of vibration-based damage identification methods. *Shock and Vibration Digest*.

[4] Farrar CR, Worden K (2007). An introduction to structural health monitoring. *Philosophical Transactions of the Royal Society A: Mathematical, Physical and Engineering Sciences*.

[5] Sohn H, Farrar CR, Hemez FM, Shunk DD, Stinemates DW, Brett RN (2003). A review of structural health monitoring literature: 1996–2001.

[6] Worden K, Dulieu-Barton JM (2004). An overview of intelligent fault detection in systems and structures. *Structural Health Monitoring*.

[7] Farrar CR, Doebling SW, Cornwell PJ, Straser EG Variability of modal parameters measured on the Alamosa Canyon Bridge.

[8] Lingras P (1998). Comparison of neofuzzy and rough neural networks. *Information Sciences*.

[9] Ahn BS, Cho SS, Kim CY (2000). Integrated methodology of rough set theory and artificial neural network for business failure prediction. *Expert Systems with Applications*.

[10] Xiang X, Zhou J, Li C, Li Q, Luo Z (2009). Fault diagnosis based on Walsh transform and rough sets. *Mechanical Systems and Signal Processing*.

[11] Tay FEH, Shen L (2003). Fault diagnosis based on rough set theory. *Engineering Applications of Artificial Intelligence*.

[12] Zhou R, Yang JG (2006). The research of engine fault diagnosis based on rough sets and support vector machine. *Transactions of CSICE*.

[13] Li JR, Khoo LP, Tor SB (2006). RMINE: a rough set based data mining prototype for the reasoning of incomplete data in condition-based fault diagnosis. *Journal of Intelligent Manufacturing*.

[14] Geng Z, Zhu Q (2009). Rough set-based heuristic hybrid recognizer and its application in fault diagnosis. *Expert Systems with Applications*.

[15] Jensen R, Shen Q (2004). Semantics-preserving dimensionality reduction: rough and fuzzy-rough-based approaches. *IEEE Transactions on Knowledge and Data Engineering*.

[16] Hu Q, Yu D, Liu J, Wu C (2008). Neighborhood rough set based heterogeneous feature subset selection. *Information Sciences*.

[17] Düntsch I, Gediga G (1998). Uncertainty measures of rough set prediction. *Artificial Intelligence*.

[18] Pawlak Z (1991). *Rough Sets: Theoretical Aspects of Reasoning about Data*.

[19] Li R, Wang Z-O (2004). Mining classification rules using rough sets and neural networks. *European Journal of Operational Research*.

[20] Bazan J, Skowron A, Synak P Dynamic reducts as a tool for extracting laws from decisions tables.

[21] Craven MW, Shavlik JW (1994). Using neural networks for data mining. *Future Generation Computer Systems*.

[22] Lu H, Setiono R, Liu H (1996). Effective data mining using neural networks. *IEEE Transactions on Knowledge and Data Engineering*.

[23] Swiniarski RW, Hargis L (2001). Rough sets as a front end of neural-networks texture classifiers. *Neurocomputing*.

